# Human rights in childbirth, narratives and restorative justice: a review

**DOI:** 10.1186/s12978-016-0264-3

**Published:** 2017-02-02

**Authors:** A. U. Lokugamage, S. D. C. Pathberiya

**Affiliations:** 1 International MotherBaby Childbirth Organisation (NGO), London, UK; 2 Advisory Board of Human Rights in Childbirth (NGO), London, UK; 3 Trustee Evelyn Oldfield Unit (NGO), London, UK; 4 Whittington Hospital, Department of Women’s Health, University College London, Jenner Building, Magdala Avenue, London, N19 5NF UK

**Keywords:** Narratives, Human rights in childbirth, Restorative justice, Obstetric violence, Respectful maternity care

## Abstract

This review describes the emerging global debate on the role of human rights childbirth. It is also tailored to a UK perspective in view of the *Montgomery v. Lanarkshire* [2015] legal ruling and it implications to practice. We can never underestimate the power of humane care on health. The compassion and evidence based medicine agenda in healthcare is interconnected with human rights in healthcare, feeding into the principles of decision making and patient centred care. When this has not happened and there is been healthcare conflict, the power of storytelling serves to connect disparate parties to their common humanity. Narratives are an important aspect of restorative justice processes and we suggest that this could be beneficial in the field of human rights in childbirth.

## Plain english summary

This article looks at human rights in the area of maternity care, following a recent UK legal case. In the past it used to be that a doctor tells the patient what to do and the patient had no choice but to follow. Now, it is not enough for doctors, midwifes, nurses and others to recommend treatment, even though they may look at the scientific evidence when advising medical care. They must also listen to the women/patients, and hear what they want and don’t want. Treatment that is both compassionate and based on medical evidence is connected to human rights in general, and should be taken in to account when decisions are being made about care. We are saying that in those situations where patients are not listened to and there is a negative effect, storytelling can be used to bring together people, so each party understands the other person’s viewpoint.

## Background

Human rights in childbirth is an emerging field within reproductive health rights. The Millennium Development Goals of the United Nations (UN) have drawn to a close and so far there had been a focus on improving maternal health within the context of improving access to facility based healthcare, but what is becoming clearer is that this alone is not sufficient [1, 2]. Although the previous focus has been the equal and fair access to healthcare, there is now a greater recognition of the importance of dignity, respect and autonomy for women who do utilise healthcare facilities [3]. The FREDA principle, is a useful human rights summary of the core issues at stake - fairness, respect, equality, dignity and autonomy [4]. The new UN Sustainable Development Goals are far more rights based.

This review is relevant at this particular time in the history of maternity care, because as eloquently put by grass roots activist, Milli Hill, founder of the Positive Birth Movement “In spite of the huge appetite for positive change, there is still a huge amount of polarity in the birth world. Women versus the system. Midwives versus obstetricians. Holistic midwives versus obstetric midwives. Doulas versus doctor etc. This polarity does not create a great environment for women to give birth in. Trust becomes lacking or lost. I see this all the time, especially on social media - women, doulas, midwives etc., versus the system. This does not improve safety, and it does not make for full freedom of choice. I’d like to urge everyone today to help us to move the emotion and the language away from this polarity....Let’s build bridges today, not walls!” [5]. So, the purpose of this review is to draw together and weave relevant ideas which have pre-existed in the field of medical humanities and law into a plausible model of bridge building for respectful maternity care.

### Human rights in the global birthing arena

Women who receive care from factory line conditions within health facilities are experiencing disrespect and abuse worldwide. Factory line conditions includes care which denies dignity, privacy, respect for autonomy to the patient such as where women are made to adhere to routine protocols without consent i.e., to lie on delivery tales for hours without freedom of movement, forced to give birth while lying flat on their backs or in stirrups, routinely administering intravenous lines without medical need and episiotomies as of routine [6, 7]. Their rights are denied in relation to: decision making over their physical integrity, self-determination, privacy, family life and spiritual freedom. This phenomena has been noted in the WHO statement on ‘Prevention and elimination of disrespect and abuse during childbirth’ [3], which states “*many women experience disrespectful and abusive treatment during childbirth in facilities worldwide. Such treatment not only violates the rights of women to respectful care, but can also threaten their rights to life, health, bodily integrity, and freedom from discrimination. This statement calls for greater action, dialogue, research and advocacy on this important public health and human rights issue*”.

‘Human Rights in Childbirth’ (HRIC) a Hague-based non-governmental organisation has been prominent from a consumer perspective in advocating for the rights of birthing women. The humanisation of childbirth movement in Brazil has been lobbied for, by Brazilian organisations such as ReHuNa - Rede pela Humanização do Parto e Nascimento (Brazilian Network for the Humanization of Childbirth) and Parto do Princípio - Mulheres em Rede pela Maternidade Ativa (Start from the Beginning, Women Networking for Active Maternity). The White Ribbon Alliance (WRA) Global Respectful Maternity Care Council consists of more than 200 organizations and individuals, globally. WRA and USAID’s Traction project are spear heading the ‘Respectful Maternity Care’ (RMC) campaign to which the International Federation of Gynecology and Obstetrics (FIGO) and the International Confederation of Midwives (ICM) have lent their support. The International MotherBaby Childbirth Organisation (IMBCO) has also developed the 10 steps International MotherBaby Initiative (IMBCI) which contain recommendations for rights based optimal maternity care and have network of collaborating sites [8, 9]. The IMBCO has developed a validated Women’s Questionnaire as a tool to assess the extent of human rights practices in maternity facilities [8, 10]. In the UK, the charity Birthrights works to improves women’s experience of pregnancy and childbirth by promoting respect for human rights.

The WHO has recognised that childbirth has become over-medicalised particularly in the case of low risk pregnancy and that the caesarean section rate worldwide is much higher than it needs to be [11]. The over-medicalisation of childbirth without informed consent has been also termed from a human rights perspective as ‘Obstetric Violence’. This term was first officially formulated in 2007 when it was introduced in Venezuela as a new legal term [12]. A definition of ‘Obstetric Violence’ is *“the appropriation of the body and reproductive processes of women by health personnel, which is expressed as dehumanized treatment, an abuse of medication, and to convert the natural processes into pathological ones, bringing with it loss of autonomy and the ability to decide freely about their bodies and sexuality, negatively impacting the quality of life of women”* [13]. Amnesty International (Uruguay) has made a powerful documentary film on Obstetric Violence, which is available on YouTube and is a reflective teaching aid for all those involved in maternity care [14]. In the United States of America, the *Amicus Curiae* Brief of HRIC, *Dray v. Staten Island University Hospital* [15], describes human rights violations due to the over-medicalisation of maternity care.

The emerging debate on the recognition of the role of women’s human rights in childbirth rests on the core issues of women’s autonomy over their health, as well as access to health care systems that treat them with dignity and respect [1]. These are all contemporary aspects of patient experience and public engagement in the birth arena. Indeed social media is being increasingly used by patients as a platform for exchanging views, lobbying and conflict whereby changing the previous power equilibrium in the relationship between patients and healthcare providers [16, 17].

In the UK, the Francis Report [18] highlighted more generic human rights violations due to industrialised healthcare systems driven by the pursuit of hospital economic targets. In the specific case of Mid Staffordshire Hospital, the Francis Report found a story of “*appalling suffering of many patients. This was primarily caused by a serious failure on the part of a provider Trust Board. It did not listen sufficiently to its patients and staff or ensure the correction of deficiencies brought to the Trust’s attention. Above all, it failed to tackle an insidious negative culture involving a tolerance of poor standards and a disengagement from managerial and leadership responsibilities. This failure was in part the consequence of allowing a focus on reaching national access targets, achieving financial balance and seeking foundation trust status to be at the cost of delivering acceptable standards of care.*” In the provision of maternity care, public interest has arisen in provider behaviours in the context of human rights, so when this is juxtaposed against a very inflamed feminist agenda, there is great potential for conflict. There is a long history regarding the balance of female power versus patriarchal systems and particularly in childbirth. The ‘inflamed agenda’ is well depicted in Olorenshaw’s article in The Huffington Post ‘Feminism Has Focused On The Boardroom But It Is Time To Remember The Birthing Room‘[19], which describes one particular ‘Women’s Voices Conference’ but the strength of feeling could be equally applied to numerous Human Rights in Childbirth conferences too. The persistence of androcentric influence despite the increase in female obstetrician numbers has been noticed too [20–24]. Most of the publications on this debate have not been in the ‘obstetric press’ and therefore many obstetricians may be unaware of it. Indeed in a recent British legal case, *Montgomery v Lanarkshire* [25] where a woman sought medicalised childbirth instead of natural childbirth, the court recognised the historical theatre of paternalism within the obstetric profession - “*social and legal developments which we have mentioned point away from a model of the relationship between the doctor and the patient based upon medical paternalism*”. Essentially now the doctor is under a duty to take reasonable care to ensure that the patient is aware of any material risks involved in proposed treatment, and of reasonable alternatives. A risk is “material” if a reasonable person in the patient’s position would be likely to attach significance to it, or if the doctor is or should reasonably be aware that their patient would be likely to attach significance to it. This strengthens women’s rights over their autonomy and bodily integrity, either for or against medical interventions and highlights the importance of patient centred care. So instead of a vicious cycle of misunderstanding between technocratic medical organisations on the one hand and feminists and/or healthcare human rights groups on the other, we ask the question in this article, ‘how can we transcend this conflict?’ This paper draws upon some pre-existing arenas of storytelling within medicine, discusses their benefits in order to ask whether restorative justice and its story telling component could be an important bridging point to resolution of conflict in human rights in childbirth? This extends to other areas of healthcare.

### Guidelines, compassion deficit, the narrative and story telling

#### Limitations of evidenced based medicine

Evidence based medicine (EBM) was a movement that led ‘medicine’ away from the philosophy authoritarian practice as the norm, to one which was influenced by available research findings. It is common viewpoint for physicians to think that often the practice of medicine is very scientific and objective [26]. However not all decisions made by doctors are rational, logical and grounded in science. Doctors’ beliefs and preferences also influence decisions [27]. Prof. Trisha Greenhalgh and others argue that it has “*become subtler and harder to detect*” evidence bias and the vested interests [28]. Analysis of US and UK Obstetrics and Gynaecology guidelines reveals that only the minority of recommendations are based on high quality, consistent evidence [29, 30]. This is why in areas of scientific uncertainty it is very important that there is collaborative decision making which is a cornerstone of patient centred care.

Contemporary healthcare is now being driven by a technocratic model where complex health, social, political and economic elements are protocolised, guided by risk, cost and fear, at the expense of personalised care. Accordingly, patients can feel *“tyrannised when their clinical management is inappropriately driven by algorithmic protocols, top-down directives and population targets.”* [28] Consequently, in some cases, evidence based medicine can be a shackle to a woman’s autonomy. Greenshalgh et al. calls to *“individualise evidence and share decisions through meaningful conversations in the context of a humanistic and professional clinical-patient relationship”* [28]. The Evidenced Based Medicine Renaissance Group, have coined this development with the term ‘#realEBM’ [28] which has a Twitter following. Indeed there are parallels between the ‘#realEBM’ movement, the ‘preventing over-diagnosis’ movement [31], the ‘dangers of too much medicine’ movement [32] (extensively published in the British Medical Journal) and groups that point to the over-medicalisation of childbirth [33–36].

The UK’s National Institute of Clinical Excellence December 2014 guideline on intrapartum care for healthy women and babies [37] and particularly place of birth, is an important document in changing this pattern of over medicalised birth. But this guideline again should not be applied in a ‘one size fits all’ manner. Nuanced, humanised, patient centred care is key to the application of evidence base medicine in a rights based approach. The commonly used term ‘shared decision making’, may not be correct in the context of human rights, as the health provider can share the information but the decision is ultimately the patient’s [38]. This is “*because a patent would consider other factors such as quality of life in addition to medical expert opinion when deciding on a course of action”* [39]. *Montgomery v Lanarkshire* [25] ruling in the UK shows that human rights cuts both ways for over medicalised versus medicalised birth. Patient centred care is hugely important.

### Evidence of improving healthcare outcomes with compassion

Sometimes health providers simply do not realise that they have lost their compassion through insensitivity caused by working in some healthcare systems. Prof Louise Aronson, medical educationalist with special interest in reflective medicine and narrative based medicine, has observed *“We doctors do many things that are otherwise unacceptable. We are trained not only in how to do such things but in how to do them almost without noticing, almost without caring, at least in the ways we might care in different circumstances or settings”* [40]. This can also true for the nursing and midwifery profession and therefore it is important to avoid unintentional blindness of any health provider to dehumanised aspects of industrialised healthcare [41]. Cochrane reviews on continuity models of midwife led care and continuous emotional support in labour clearly demonstrate that humane relational maternity care trumps technocratic care in creating safe childbirth outcomes which cost less [42–45].

In Dr Robin Youngson’s book *Time to Care* [46], he discusses compelling health economic evidence about the critical importance of compassion in healthcare. For instance compassionate, whole-person care in terminal lung cancer such as early access to palliative care leads to less depression and longer survival [47]. Also diabetic patients of high-empathy primary care physicians had 42% fewer hospital admissions for metabolic crisis than patients of low-empathy physicians [48]. In addition, Youngson clearly demonstrates that healthcare worker burnout through working in dehumanised industrialised healthcare conditions leads to lack of compassion towards patients [46, 49].

*The Lancet*’s 2014 Midwifery Series [50] notes that industrialised maternity services that have deficits in provision of compassionate care, are not only because of a lack of training but also due to discrimination and abuse that is linked to, and reinforced by, systemic conditions, such as degrading, disrespectful working conditions and multiple demands, and can be seen as a signal of a “health system in crisis” [51]. With such complexity would it be prudent to explore the perspective of not only women/patients but also health providers, in which storytelling may have its role to play?

### Story telling

*“Stories may not provide all the answers, but what is gained through their telling is important for social justice and democracy. They connect us to issues and to one another through the power of a narrative and the experience of empathy”* [52]. The Women’s Human Rights Storytelling Collaboratory is an interesting example of this as their platform for story-sharing was showcased at the Commission on the Status of Women (CSW59) Conference 2015 at the United Nations. They describe their method as an intense pressure cooker for catalysing analysis, learning and greater collective action in women’s rights [53]. Several healthcare crises within the National Health Services have highlighted the importance of health care providers seeing the delivery of care through the eyes of patients. The NHS England, *Compassion in Practice – One Year On* document states *“The Francis Report, the Keogh Report, the Cavendish and Berwick Reviews have all highlighted how we need to improve and in doing so have emphasized the centrality of compassion in the care we deliver. We can never be complacent and must continue to listen to the people we care for and to staff who are responsible for that care so we can continually improve”* [54]. An example of an educational tool exemplifying these principles is ‘Footprints of Birth’ [55] where women’s narratives were heard in a documentary and a further film bearing the voices of hospital staff and students demonstrated institutional listening and response to the women’s stories. Story telling can be very effective at healing health care systems that are broken. This narrative based approach is healing for the victims, but can be transformational for health care providers as seen in compassion Schwartz Center rounds [56, 57] and Balint groups [58, 59]. These are confidential healthcare professional forums that allow reflection on the emotional and social challenges of work. Through staff stories regarding clinical care surrounding demanding situations, the narratives create an empathic understanding about themselves and their own colleagues which can spill over to generate compassion for their patients. From an organisational development perspective expanding the utilisation of these narrative modalities within maternity care services may help to improve negative work place behaviours particularly in light of the UK’s General Medical Council’s National Training Survey 2014 on bullying and undermining experienced by junior doctors. The undermining behaviours were described as receiving belittling or humiliation and threatening or insulting behaviour. This document indicated that obstetrics and gynaecology, as a speciality, seems to be less supportive and had more undermining behaviours than other specialties [60]. The Royal College of Obstetricians and Gynaecologists with the Royal College of Midwives have developed a toolkit to improve workplace behaviours [61], however this is mainly trainee doctor focussed rather than encompassing the whole system such as to unearth undermining received by any staff member (senior or junior) due to the system issues found in the Francis report [18]. Furthermore, there is evidence that a significant number of consultants in obstetrics and gynaecology also experience bullying. These have been described as persistent attempts to belittle and undermine an individual’s work; undermining an individual’s integrity; persistent and unjustified criticism and monitoring of work; freezing out, ignoring or excluding and continual undervaluing of an individual’s effort. Perpetrators can be lead clinicians, clinical directors, clinical secretaries, career grade doctors, patients, administration managers, general practitioners and board-level executives [62]. So, there is clearly an endemic system problem for women, midwives and doctors.

Storytelling overlaps with the strengths of narrative based medicine which has been described as having four genres: patient stories; physician stories; narrative about physician-patient encounters; and grand narratives of sociocultural understandings of the body in health and illness. All of which have the healing potential to help parties from opposing views become involved in developing their human potential through their common humanity [63]. We can never underestimate the power of humane working conditions and humane care on health. Where there has been healthcare conflict, the power of storytelling serves to connect disparate parties to their common humanity – which would suggest that restorative justice processes could be beneficial in human rights in childbirth.

### Restorative justice

Restorative Justice (RJ) is a narrative process whereby the parties to a dispute, conflict or crime are brought into communication in order to find a way to move positively forward and build relationships. While RJ has been used in many societies of old [64], it was most notably used by the Māori in New Zealand [65]. In post apartheid South Africa, RJ was used by the Truth and Reconciliation Commission where it was absolutely key to a reduction in civil animosity [66] and more recently in UK [67], where courts have the power to defer passing of sentence post-conviction [68].

The flexibility of RJ offers great freedom to its facilitators and participants enabling them to adapt it to suit the needs of a given situation. RJ based models or programs range from a simple apology, to meetings involving stakeholders overseen by a trained moderator. RJ circles (where participants narrate a story) or Group Conferencing (discussion with all parties including community) may be suitable in addressing the concerns in health care where patients feel they have not received the care they should have.

The key objectives of the process would be to repair the harm suffered by the victim; person at fault becomes aware of that his actions are unacceptable and the effect his actions are having on the victims and community; acknowledging responsibility for actions; participate in reparation decision making moving forward; participation of community; and victim brought to understand the position of the other parties [69]. A successful RJ program in a hospital setting would aim to understand the aetiological factors which produce negative outcomes such as stress, lack of resources (time, training) as well as engage with patients and community in humanistic way to understand their concerns. RJ based models have the capacity to reduces health costs [70], for example in post traumatic stress disorder and as well as resolve organisational conflict. It may also become a positive influence in marketing the hospital in the community through word-of-mouth recommendations and also increase patient retention. Participants should not be limited to medical professionals and include administrative and managerial actors in health care institute [25]. This is because the issues are not merely individual but are institutional and even cultural and political in nature.

Litigation may be used by aggrieved parties in order to get monetary compensation, especially when long-term care is needed following a disability, as with *Montgomery v Lanarkshire*. [25]. However there is opportunity for RJ to assist in to moving forward amicably rather than from an aggrieved stance. Such instances may even lead to cases being resolved out of court, reducing litigation costs as well as giving insight in how to improve systems of care [71].

In *Montgomery v Lanarkshire* [25] a doctor chose to omit giving information on risks on planned procedures to a patient which would have enabled the patient to make an informed decision. The court found that there was a deficiency of honesty from the onset of the relationship between the patient and the doctor. As well as a lack of respect for the patient’s autonomy, it also led to a negative outcome.

Limitations in patient centred care and consultation conditions, whether concerned with short consultation time when trying to fit in counselling about rarer outcomes; or a deficit in narrative based medicine; or pressures on health care providers which reduce empathic consultations that can pick up on patient preferences; or a deficit of shared decision making, are all nuanced interactions that are highlighted in the *Montgomery case* [2015]. The court’s decision is subject to the availability of this information and also the prosecution’s skill in presenting arguments. This type of legal discrepancy may have resulted in the seemingly two opposing rulings from the European Court of Human Rights (ECHR) about the rights for women to have home birth *Tzernovsky v. Hungary* [72] and *Dubska v. Czech Republic* [73, 74]. Both these ECHR cases have generated a huge amount of conflict and debate between human rights and feminist groups against European technocratic maternity systems in Hungary and the Czech Republic.

## Conclusions

In this review, we have given an overview of human rights in childbirth, looked at rights-based individualised decision making in a compassionate model of evidence based medicine, highlighted the assets of narratives in medicine and pointed to the benefits of restorative justice. Human rights in childbirth, has served as a forum for highlighting many untapped or repressed areas of rage, anger and conflict within maternity care. Will this contemporary form of feminist rebellion against dehumanised healthcare lead to transformation of institutional attitudes? Perhaps restorative justice can transmute the polarised views that that can be created in human rights in childbirth disputes? The main reasons for RJ’s popularity and effectiveness is the restorative processes’ ability to build better relationships and strengthening communities, while being able to collectively discover a way to move forward by resolving conflict and healing harm. The movement forward is a joint effort that all stakeholders are contributors. For this reason restorative justice processes instead of litigation may break a cycle of animosity, defensive medical practice and post traumatic stress which litigation can amplify [70].
